# Placental transmogrification of the lung presenting as a consolidative lesion with bronchiectasis

**DOI:** 10.1111/1759-7714.13066

**Published:** 2019-06-25

**Authors:** Min Kyun Kang, Do Kyun Kang, Youn‐Ho Hwang, Ji Yeon Kim

**Affiliations:** ^1^ Department of Thoracic and Cardiovascular Surgery Inje University College of Medicine Busan South Korea; ^2^ Department of Pathology, Haeundae Paik Hospital Inje University College of Medicine Busan South Korea

**Keywords:** Consolidation, lung pathology, placenta

## Abstract

We report a case of a 37‐year‐old man with a persistent consolidative lesion in the right lower lobe. A chest computed tomography scan showed a 2 cm focal consolidative lesion in the lateral basal segment of the right lower lobe. The size of lesion had slightly increased over four months. Wedge resection of the right lower lobe was performed with the intention to diagnose the lesion. Pathological examination showed placental transmogrification of the lung. We describe this rare lung disease, which presented with the unusual radiologic findings of a consolidative lesion. Placental transmogrification of the lung should be considered in the differential diagnosis of pulmonary consolidative lesions.

## Introduction

Placental transmogrification of the lung (PTL) is a rare benign disease, which was first described in 1979 as a subtype of bullous emphysema.^1^ However, PTL does not have any placental properties. The name of this disease comes from its morphology, which is similar to that of the placenta. A microscopic view shows that it is filled with papillary structures covered by epithelial cells.^2^ Its etiology and pathogenesis is unknown, although there are a few reports that support congenital origin.^3^ To date, 34 cases have been identified in English literature.^4^ PTL usually presents as bullous lesion, and rarely as cysts or nodules on radiologic imaging.^5^ This report describes a case of PTL with unusual radiologic findings, which was treated by surgical resection.

## Case report

**Figure 1:**
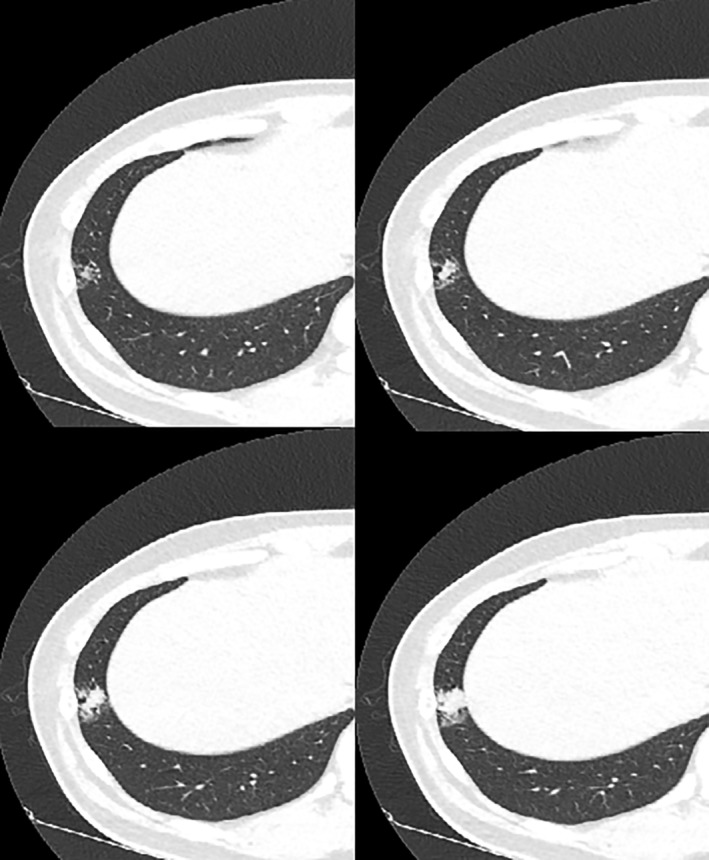
A preoperative chest computed tomography scan shows a focal consolidative lesion with bronchiectasis in the subpleural region of the lateral basal segment of the right lower lobe.

**Figure 2:**
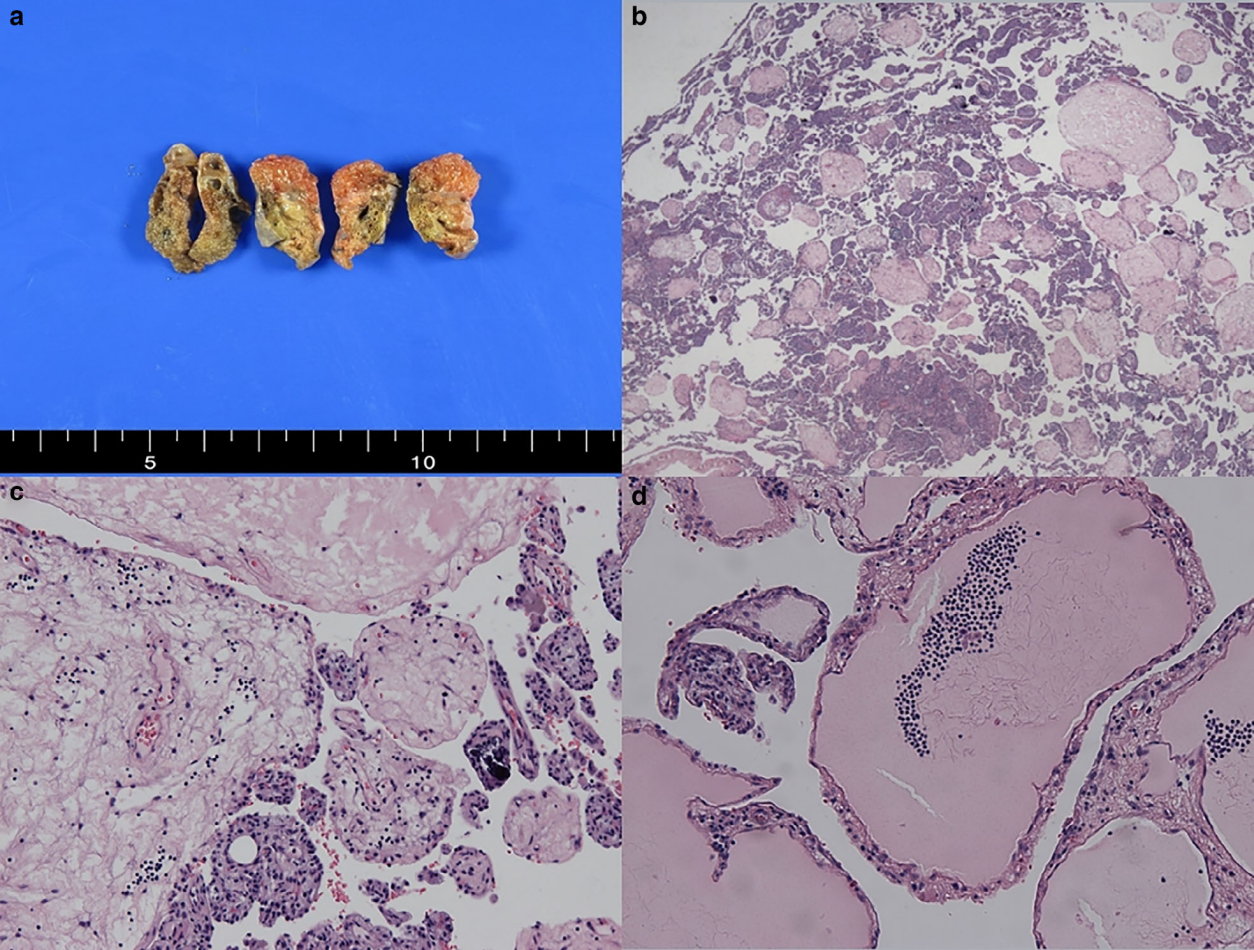
(**a**) Serial sections of lung tissue show an ill‐defined mass with a red, bosselated cut surface. (**b**) Microscopic examination of the mass revealed multiple papillary processes with a histologic resemblance to chorionic villi of the placenta. On high power microscopic view, the papillae of various sizes were edematous and lined by focally hyperplastic pneumocytes. (**c**) Calcifications were frequently noted. (**d**) Some cores of the papillae contained dilated lymphatics.

A 37‐year‐old man presented to the thoracic surgery department with a persistent consolidative lesion in the right lower lobe. He was a 14‐pack‐year smoker but denied a history of recent trauma or significant disease. A chest computed tomography scan revealed a 2 cm focal consolidative lesion with bronchiectasis in the subpleural region of the lateral basal segment of the right lower lobe. The patient had been under observation for approximately four months in the outpatient pulmonology department. The size of lesion had slightly increased over this time (Fig 1). No lymphadenopathy or other mass lesions were identified. The patient underwent single port video‐assisted thoracoscopic surgery. Wedge resection of the lesion was performed and showed an ill‐defined fungating mass with focal cystic change, measuring 7.5 x 6.0 x 1.5 cm and weighing 410 g. Gross and microscopic features of the lesion are shown in Figure 2. These findings were consistent with the characteristics of PTL. The patient had an uneventful clinical course and was discharged without complication.

## Discussion

PTL is referred to as a placentoid bullous lesion because the most common radiologic finding is a bullous emphysema pattern. Cystic lesions or solitary nodule patterns are rarely observed.^2^, ^5^ Patients may be asymptomatic or are diagnosed with dyspnea, chest pain associated with pneumothorax, or chronic obstructive lung disease as a result of bullous emphysema. Papillary structures covered by hyperplastic pneumocytes are observed on hematoxylin and eosin stain. The immunohistochemical staining pattern is positive for CD‐10 and vimentin and negative for CK, desmin, S100 protein, and smooth muscle actin. In 33 of the 34 cases in the literature, surgical resection was the treatment choice. Surgical options included wedge resection (*n* = 10), lobectomy, (*n* = 10), and pneumonectomy (*n* = 7) according to the distribution and size of the lesion. A surgical option was not mentioned in six cases.^4^ Thus, surgical resection is the treatment of choice, and was curative and successful without recurrence in the cases discussed in the literature. Our case was unique because the 37‐year‐old patient presented with unusual radiologic findings of a consolidative lesion with bronchiectasis, which was not observed in any of the previously reported cases. The treatment is consistent with those of previous cases in the literature and achieved a satisfactory outcome. Although it is a rare disease, our report serves as reminder that PTL should be considered in the differential diagnosis of a consolidative lesion with or without bronchiectasis. PTL is best treated by surgical resection.

## Disclosure

No authors report any conflict of interest.
